# Supplementary material for: Motion sickness and sense of presence in a virtual reality environment developed for manual wheelchair users, with three different approaches
Source: PLoS One. 2021 Aug 19;16(8):e0255898. doi: 10.1371/journal.pone.0255898 (PMC8375983; doi:10.1371/journal.pone.0255898)
Supplement: Attachment — Submitted filename: Review Feedback_ Motion sickness and sense of presence in three virtual reality environments developed for manual wheelchair users.pdf [file pone.0255898.s001.pdf]

The results of this study rely on there being no difference between MSAQ and IPQ results between the VR systems used. If there were significant differences between these systems, then the data would mandate an entirely different set of analyses than what was used in this study. However, the authors state their comparison across VR training systems is underpowered which makes a comparison moot. The authors need to increase the sample size of this study so that their comparison across VR systems is not underpowered and they can have statistical confidence in their results As it stands, the underpowered result cannot be trusted as indicative one way or another that VR systems were no different in MSAQ and IPQ. Failing to reject the null hypothesis should not be taken as evidence of the null hypothesis. Without this additional data, the findings of this study are equivocal.

Additionally, the authors claim that training sessions reduced MSAQ scores. However, training sessions was not the only independent variable in the study. Exposure to VR system type is a second independent variable which was not intended by the authors. This study would benefit from a different methodology that evaluated only one type of VR system. As the study stands, it's impossible to say if training session alone led to reduced MSAQ scores. Perhaps it was changing VR systems across participants which led to reduced MSAQ scores. Perhaps it was both VR system type exposure, and repeated exposures (training sessions) which led to the reduction in MSAQ scores. The methodology and underpowered comparison of VR systems MSAQ and IPQ scores makes this impossible to determine.

In line 83 the author refers to fake movements in a user's peripheral vision. This is unclear. The author should provide an explanation of what a fake movement is or means.

In lines 159 the authors have a typo, "arder"

Unable to understand what the preconditions -3 to 0 trials are. The authors should provide more information about what users did during these training sessions.

Lines 214-215 the authors say, "the statistical procedures are selected in a way to maximize the power. Therefore, wherever possible, the parametrics methods used that have higher statistical power than non-parametric methods. However if the assumptions were not met, the non-parametrics methods have been utilized."

- This is confusing. The authors should be using analyses that meet the constraints of the statistical assumptions. If this is what the authors did, I suggest rewriting this passage to simplify it. The authors can simply say they used parametric and nonparametric tests when appropriate.

The justification for not using a Bonferonni correction, or any other statistical correction for multiple comparisons, is untenable. The decision to correct for multiple comparisons cannot be made on the basis of what is convenient for the study or results. The authors state that because they're looking for similarities as opposed to differences, corrections for multiple comparisons aren't necessary. The opposite is true. The authors should use conventional

statistical methods (i.e. multiple comparisons corrections) which adhere to widely accepted and established statistical practices to demonstrate any claims they make based on their data.

The authors should report the mean and standard deviation for MSAQ scores, and should report the sample size, the p value, and F value for the MANOVA.

Lines 288-294 editorialize in the results section. The authors should plainly state their result, and expand upon the importance of the result in the discussion section.

Line 445-447 The authors state that some results of the study could not be confirmed statistically. However, a result cannot exist without empirical evidence. The authors claim here that a result exists in which they have no evidence for. The authors should remove this sentence.

Lines 414-425 The authors state that there was no significant difference in MSAQ or IPQ for any VR system, but then state that a larger sample size would be needed to identify significant results. The authors then claim that future work doesn't need a larger sample size. This is problematic for two reasons. The authors specifically identify that their null result in MSAQ and IPQ across systems is likely due to too small a sample size. Because this is the case, the authors should not claim there is or is not a difference between VR training systems. A second concern is that the authors say future work should not focus on getting a large enough sample size to identify statistical differences between the systems. However, this would leave future researchers in the same bind the current authors are in; their analyses are underpowered and cannot be used to validate their claims one way or another.
