# Supplementary material for: Motion sickness and sense of presence in a virtual reality environment developed for manual wheelchair users, with three different approaches
Source: PLoS One. 2021 Aug 19;16(8):e0255898. doi: 10.1371/journal.pone.0255898 (PMC8375983; doi:10.1371/journal.pone.0255898)
Supplement: Attachment — Submitted filename: Response to Reviewers.docx [file pone.0255898.s003.docx]

**Motion sickness and sense of presence in a virtual reality environment developed for manual wheelchair users, with three different approaches**

Zohreh Salimi, Martin Ferguson-Pell

Dear Dr. Stoffregen,

Thank you for sending us the letter of review for the above paper on May 17, 2021, and also for giving us the opportunity to send a revised version of our manuscript. We also would like to thank the reviewers for their comments.

We have applied the reviewers’ comments and addressed them in the below table. The revised manuscript is uploaded alongside this letter (one with changes tracked and one with all revisions applied and accepted). I hope that the revised version is acceptable for publication in Plos One.

Please do not hesitate to contact me if I can provide any further information about this study.

Sincerely yours,

Zohreh Salimi

|  | Comments | Responses |
| --- | --- | --- |
| Reviewer #2 | I want to thank the author for responding to/incorporating my feedback. I still think the underpowered analysis is problematic, but not fatal to the paper. | We are happy to read that you feel more confident about the paper. |
| Reviewer #3 |  |  |
| Overall | Thank you for the revisions you made and providing a rationale for the items that you left from the original submission. Unfortunately, there are still some significant items that need to be addressed in the introduction and in your results/analysis. | We are happy to address them. Thank you for your constructive approach. |
| 1 | Your introduction is a general statement of the problem of VIMS in VR followed by a research example prior to discussing your work in the manuscript. The issue that the discussion of Chattha et al does not motivate or justify your manipulation/method, it is a jarring switch between the problem set-up and the overview of your project. You would be better served to address why movement/experience using the wheel chair is important (in general the connection between perception and action). | The Chattha paragraph was relocated to a more proper place (where the factors triggering motion sickness was discussed). In addition, a paragraph was added, as suggested by the reviewer, before switching to discussing our study, to facilitate a better transition:  “As mentioned, when there is a mismatch between vestibular and visual stimuli, VIMS could trigger. This is roughly whenever the user moves around, or looks around, in the virtual world. We also mentioned that VR has a potential to be used to assist with many different areas, including for training and conducting related research studies for wheelchair users, as one important part of the society who face miscellaneous difficulties on a daily basis; difficulties ranging from troubles in moving around and navigating in and outside home and accessing buildings, to secondary injuries that they usually undergo as a consequence of the above-normal and repetitive load they experience in their upper-body which they have to use for ambulation. We developed this study to simulate navigation using wheelchair in the VR, and since combination of navigation and VR has a high probability of inducing VIMS, we concentrated on mitigating VIMS while trying to ascertain higher sense of presence.” |
| 2 | In addition, your stated goals for the project (process of developing a more viable VR interaction) really don't map onto the the analyses you propose. Again, you state (as in the original) that the statistical outcomes were not the goal, rather the development of a compelling and sickness "free" virtual experience. Given this it is hard to understand the choice of analyses (more power does not help when the choice of analysis itself is non-appropriate for the stated goal). It appears that you are asking for a perceptually ranked ordering of the VR systems, again non-parametrics are tailored for this type of analysis. MANOVA is not designed to tell 'optimum' or 'best', just differences and it is not a 'solution' for dealing with small samples sizes (in fact it works much better with larger samples). Adjusting the frame of your introduction and analysis may solve these issues. | We understand that the wording in the literature, “statistics are not the main focus of the study” has made you uncertain about why then we have made many difficult statistical round-abouts to deal with the data. Thus, we have revised that paragraph and tried to explain our approach better in the introduction, as below:  “We also wish to note that although Statistics was not the focus of this research study, the statistical approaches were selected with care rather than to simplify the analyses. In order to get the most rigorous results, we carefully assessed each outcome for the six analyses to determine which of the parametric or nonparametric methods would suit the conditions of that dataset and chose the proper statistical approach accordingly”.  In case you still are not very sure about the rational behind choosing the statistical approaches in this manuscript, we have provided more explanations in the following:  It seems that the point you are raising is more about “types of statistical analysis” e.g., difference-finding, similarity-finding, or optimum finding, than using parametric or non-parametric approaches, because you can perform all those analyses using both parametric and non-parametric methods, where conditions (e.g., normal distribution) are met. In this manuscript, we have used both methods, where appropriate, to see if the systems are different, and if yes, which of the 3 systems are better than the others; a normal and very frequent use of MANOVA in the literature.  As reported frequently in the literature, e.g., Hopkins (2018)^[1]^, parametric methods should be used when we have continuous data that is normally distributed, and non-parametric methods should be used when we have categorical data, or we do not have normal distribution (*exactly* what we did in this manuscript). Despite what you are suggesting, it is *never* advised in the literature to use nonparametric methods when we have normal distribution (mainly our case).  I am not getting different type of the results from the two methods. Even with the non-parametric method, Kruskal-Wallis, I report the significance level, and based on that, I judge if there was a meaningful difference- just as what we did with the MANOVA.  Both parametric and non-parametric methods work better with larger sample sizes. That is true. But for a given fragile sample size, and when I get the same information from running MANOVA or its non-parametric counterpart: Kruskal-Wallis, I would prefer to use the parametric method rather than use a less powerful method that does a similar job.  Finally, mixing parametric and non-parametric methods is not unprecedented in the literature. For example, the following has used both parametric and nonparametric tests in the same study, as per assumptions were satisfied, just like what we did in our study:  “Niksirat, K.S., Silpasuwanchai, C., Ahmed, M.M.H., Cheng, P., Ren, X., 2017. A framework for interactive mindfulness meditation using attention-regulation process. Conf. Hum. Factors Comput. Syst. - Proc. 2017-May, 2672–2684. https://doi.org/10.1145/3025453.3025914"  With all due respect, the authors of this manuscript understand that while using the non-parametric method for all the analyses of this manuscript would keep things neat, the more rigorous way is to use parametric methods wherever the conditions are met. This strengthens the integrity of the analysis and reduces the chance of type II errors. |

1. - Hopkins S, Dettori JR, Chapman JR. Parametric and Nonparametric Tests in Spine Research: Why Do They Matter? Global Spine Journal. 2018;8(6):652-654. doi:[10.1177/2192568218782679](https://doi.org/10.1177/2192568218782679)
